# An examination of a voluntary policy model to effect behavioral change and influence interactions and decision making in the freight sector

**DOI:** 10.1016/j.trd.2016.11.018

**Published:** 2016-12-21

**Authors:** Cheryl Bynum, Chien Sze, Denise Kearns, Buddy Polovick, Karl Simon

**Affiliations:** a U.S. Environmental Protection Agency, 2000 Traverwood Drive, Ann Arbor, MI 48105, USA; b U.S. Environmental Protection Agency, 1200 Pennsylvania Avenue, N.W., Washington, DC 20460, USA

**Keywords:** Market-based approach, Voluntary policy model, Freight transportation, Carbon accounting, Emissions calculator tool, Benchmarking performance

## Abstract

Freight transportation is essential to maintaining commerce and economies in the United States and globally. However, freight transportation is known to have significant environmental and public health impacts. Harmful emissions of carbon dioxide, methane, hydrofluorocarbons, and black carbon increase the risk of global climate change. Emissions of nitrogen oxides and particulate matter contribute to serious public health risks including increased incidences of premature death, and increased severity of respiratory and cardiovascular illness. As trade is increasingly globalized and economies expand, harmful air emissions from goods movement are projected to increase at faster rates than all other sources of transport-related emissions. While mandatory rules such as advanced vehicle emission and fuel quality standards reduce emissions from new vehicles, the vast legacy fleet of heavy duty diesel vehicles present a challenge for policy makers around the world. This paper examines how a voluntary policy model, the U.S. Environmental Protection Agency’s SmartWay Transport Partnership, fosters behavior change, facilitates strategic interactions and enables more informed decision-making in the freight sector to improve performance and reduce emissions. The effectiveness of this innovative model has generated international interest and led to program replication in other countries.

## 1. Introduction

Trade growth and an ever-expanding global economy are creating an unprecedented and seemingly endless demand for freight transport capacity and infrastructure. As a result, carbon emissions from freight transport are growing at a rapid rate. Between 1990 and 2013, U.S. greenhouse gas emissions from freight transport grew by more than 50%, an increase of almost 200 million metric tons (U.S. Environmental Protection Agency, 2015a, pp. 104–105).

Projections are that by 2050, carbon dioxide emissions from global freight will nearly quadruple, rising by a factor of 3.9, from an estimated 2.1 billion metric tons (2010) to 8.1 billion metric tons (OECD/ITF, 2015). At this rate of growth, freight emissions could account for more than one-half of all transportation related carbon emissions, exceeding emission levels from personal transportation sources for the first time (OECD/ITF, 2015).

Until recently, however, the adverse, long-term impacts of global trade and commerce on growth in carbon emissions were largely ignored (Golicic et al., 2010, pp. 47–55).

As the world’s nations seek to reduce the risk of global climate change and curb harmful air emissions, there is an urgent need to address the rapid growth in freight-related emissions using all extant policy approaches.

In the U.S. and throughout the world, trucks are the single largest source of greenhouse gases from freight, accounting for over half of all trade-related carbon emissions (Miller and Facanha, 2014; OECD/ITF, 2015; U.S. Energy Information Administration, 2015). The first U.S. regulations to reduce greenhouse gas emissions and improve efficiency from freight trucks were first published in 2011 and apply to 2014 and later model year trucks (Greenhouse Gas Emissions, 2011). These regulations are projected to reduce CO_2_ emissions by about 270 million metric tons and save about 530 million barrels of oil over the lifetime of 2014–2018 trucks. In 2016, the U.S. Environmental Protection Agency (EPA) along with the National Highway and Traffic Safety Administration (NHTSA) jointly issued a second set of standards, starting in 2018 for trailers and 2021 for trucks. The new standards will cut GHG emissions by approximately 1 billion metric tons and conserve approximately 1.8 billion barrels of oil over the lifetime of the vehicles sold under the program (Greenhouse Gas Emissions, 2016).

Even as the new standards phase-in, however, millions of older, less efficient heavy-duty vehicles remain in use in the United States. This legacy fleet will continue emitting high levels of greenhouse gases and emit other harmful air pollutants well into the future. Further, few nations have yet to establish similar standards to cut greenhouse gas emissions or stringent standards to lower other harmful air pollutants from freight trucks.

Consequently, while regulations will play a critical role by addressing new vehicles, there also is an urgent and growing need for complementary policy instruments to facilitate freight sustainability. This paper examines one instrument, a voluntary policy model initiated by the U.S. EPA, the SmartWay Transport Partnership. This program can be applied to legacy vehicles and newly-efficient vehicles to further reduce emissions.

To date, little research has been done to understand the influences that lead shippers and carriers to collaborate and adopt environmental transportation practices that reduce emissions and contribute to more sustainable supply chains (Nyabusore, 2015). In this study we will examine the role of the U.S. EPA’s SmartWay Transport Partnership in facilitating such collaboration. Specifically, we will review the effect and influence of the SmartWay model on effecting behavior change and on influencing strategic interactions and decision making that result in more sustainable business practices and industry progress toward environmental goals. We will focus on three key freight sector participants: shippers (companies that use freight transportation services; represent freight demand); freight carriers (companies that provide these freight transportation services; represent supply side); and vehicle and equipment manufacturers (companies that provide transportation technologies to carriers and in some cases, to shippers).

## 2. Background

In 2004, EPA launched SmartWay Transport Partnership–a public-private initiative between freight shippers, carriers, logistics companies and other stakeholders, to voluntarily achieve improved fuel efficiency and reduce environmental impacts from freight transport. In the two years leading up to the program’s release, EPA collaborated with key stakeholders from the trucking industry and shipping community to develop the program’s structure and approaches. The concept for the SmartWay Transport Partnership was based on input from these freight industry leaders (U.S. EPA, 2004).

At that time, sustainability was an emerging concept in the corporate world. Where an awareness of sustainability existed, it typically did not extend to a shipper’s transportation operations. Transportation management was focusing on cost, speed and timeliness of delivery, equipment availability and responsiveness to change (Holcomb et al., 2014). Moreover, research shows that shipper initiatives are largely driven by company policy and that shippers tend to push sustainability requirements onto the carriers that work for them (Wolmarans et al., 2014).

Shippers attempting to include transportation in their sustainability planning, however, soon ran into a roadblock–a lack of data about the environmental performance of their carrier base. Percent reduction of carbon emissions is one of the most commonly used reporting metrics for shippers, but there was no accepted, consistent method to assess, track and verify carrier environmental performance using carbon as a metric.

Carriers are motivated to adopt sustainable business practices that will make them more competitive and help reduce costs (Wolmarans et al., 2014). Thus, carriers were driven by a profit motive to be more sustainable–by reduced fuel use, which typically correlates directly to carbon emissions reductions and costs, and a potentially a larger, more satisfied market share. However, as shipper clients began to request environmental performance data from their carriers, trucking companies were confronted by a host of new, different, inconsistent or duplicative forms and surveys from multiple customers.

The lack of uniform assessment and reporting mechanisms, and an inability to validate carrier performance data based on a consistent metric, greatly reduced its value for either shippers or carriers, in helping to effect change or influence decisions. Carriers were further confounded by a lack of reliable data on either the financial return or emissions benefit they could expect when investing in the fuel-saving technologies and pollution control equipment available to them to help meet the shipper community’s growing interest in transportation sustainability.

Notably, during the early to mid-2000s, original equipment manufacturers (OEMs) were focusing on building tractors to meet regulatory standards for criteria pollutants, specifically particulate matter and nitrogen oxides. EPA’s legal authority to regulate greenhouse gas emissions and establish carbon standards was not established until December 2009 (Endangerment Finding, 2009). Consequently, OEMs were not compelled to build tractors specifically designed to reduce fuel use and carbon emissions. Equipment suppliers, however, recognized an opportunity to develop aftermarket components that could help reduce fuel use and carbon, including aerodynamic equipment, idle reduction devices and low-rolling resistant tires. Though trucking companies and fleets recognized the potential fuel-saving and emissions benefits of these components, a trusted, credible program did not exist to verify the benefits. Thus, the market for these components was stalled.

## 3. Design of the voluntary policy model

### 3.1. Identifying market mechanisms and participant role in the freight sector

To influence behavior in the freight sector, one needs to understand the roles of each participant, the factors that influence strategic interactions among these participants, and the gaps and challenges that confound decision-making and implementation.

“Shippers” represent the demand side of freight transportation. As such, shippers make key freight decisions including:

- which modes to use;
- whether to own an in-house private fleet or hire a carrier;
- which carriers to hire and how much they are willing to pay them;
- where to source and sell goods;
- which distribution routes to use between the points of origin and destination;
- how goods are packaged and packed;
- how and when freight is picked up and delivered.

Shippers even decide which of these decisions they will make directly, and which they will outsource to a third party logistics company or 3PL. Third party logistics providers broker freight services for shipper clients which can include managing carrier procurement, distribution and warehousing–or simply handling “last mile” deliveries on a trip fee basis. From a shipper’s perspective, a 3PL functions along a continuum from consultant to carrier. From a carrier’s perspective, a 3PL functions as an intermediary to the shipper or as the shipper. For the purposes of this paper, however, we will consider 3PLs to be shippers for most of our discussion, and fold their activities into that of the shipper. Shippers have market leverage and influence, in that they may elect to hire carriers based on a wide range of factors, including cost, on-time performance, insurance, safety record, capacity commitments, certifications and environmental performance. For purposes of this paper we will consider how informing shippers of the energy and environmental efficiency of carriers can help leverage market mechanisms and drive efficiency gains.

“Carriers” move goods and represent the supply side of the freight transportation market. The decisions they make include:

- which shippers to carry for;
- how much to charge;
- how they will move the goods (some truck carriers broker rail and barge services, i.e. functioning as multimodal carriers);
- which drivers to use;
- what vehicles and equipment to buy and when;
- how those vehicles will be operated and maintained;
- whether and how they will train drivers.

Carrier does not only refer to trucking firms; it also refers to other modes of freight transportation like barge, rail, and air. However, in this paper we will use truck carriers as a surrogate for all carrier modes, since trucks are the dominant mode in the freight industry. More ton-miles of freight are shipped via truck in the United States than all other modes combined (National Highway Transportation Safety Administration, 2015). Carriers too have options in the marketplace and elect to work for shippers that suit their business models, often being selective in working with “shippers of choice” who cooperate with carriers on key business practices such as preferred loading times, driver engagement and continuity of contracts. Carriers also have tremendous purchasing power in selecting new equipment and retrofit technologies to save fuel and/or reduce emissions. Carrier acceptance and adoption of new technologies is essential to driving efficiency gains and emissions reductions from the freight sector.

“OEMs” or original equipment manufacturers, for the purposes of this paper, are the firms that design, build, market and sell new tractors and trailers. Their decisions on what to design and produce are dependent on a number of factors:

- emissions, fuel economy and GHG standards established by EPA;
- safety standards established by Department of Transportation (DOT);
- marketplace demand for durable, efficient and lowest cost equipment;
- availability of proven technologies and materials.

OEMs respond to the changing marketplace and new regulatory standards through vehicle redesigns, new equipment and other necessary modifications. They work with their suppliers and major carriers to test and demonstrate the performance of new technologies. Though OEMs have a tremendous influence in the trucking industry, they also work closely with engine manufacturers and other suppliers to adjust to market changes. A large equipment aftermarket also exists, which includes a broad range of products such aerodynamic equipment fabricators, idle reduction system providers, and tire manufacturers, among others. This paper will not dedicate analysis to the critical role played by OEMs and their suppliers; however, it is worth noting the essential role they play in the marketplace. Future research may be directed to a closer look at the role of OEMs.

### 3.2. Recognizing barriers in the freight sector

#### 3.2.1. Market barriers to technology adoption

Fuel saving technologies have been on the market for years, covering a spectrum of legitimate high quality products, as well as “snake oil” products with little or no impact. Furthermore, technology performance can vary widely depending on the application. As a result of this uncertainty, many carriers have been conditioned to avoid innovation based on variable or poor results of newer technologies. Still others are skeptical about the risks of new technologies which may not improve performance or result in breakdowns and loss of service, both of which are tremendous risks in competitive markets. Thus there is greater incentive to avoid innovation and pass the cost of inefficiency on to the shipper client via fuel surcharges. By the year 2000, a number of fuel-saving products were available in the marketplace. These included idle reducing auxiliary power units (APUs) and bunk heaters, low rolling resistance and single-wide tires, aluminium wheels, tire pressure monitoring and inflation systems, aerodynamic nose cones and trailer skirts. Investment in these technologies was relatively low with only a fraction of fleets and operators investing in them, despite manufacturer claims of fuel economy benefits. Balzaretti et al. reported that fleet operators often faced the challenge in sorting through the technologies promoted by a host of players, such as component manufacturers, resellers, and self-styled efficiency consultants (2009). Likewise, Tan and Blanco attributed to the fragmented market structure of the truck manufacturing and component industry as the primary source of obstacles to the flow of accurate and useful information (2009). Confusing economic incentives stem from complex market forces, whereby tractors and trailers which are designed and produced by different manufacturers are often owned and operated by different companies.

Furthermore, component manufacturers often operate independently of truck manufacturers, making it difficult to demonstrate and market their efficiency enhancements. And except for tire manufacturers, most trailer and component manufacturers of fuel saving equipment and devices are small or even micro businesses, making it harder for these suppliers to absorb research costs and risk producing new products that the market will not buy.

In addition, there was no industry-wide agreement on standards or methodologies for measuring the efficiency of heavy trucks, nor were individual fleet test results easily replicable. This created much uncertainty regarding payback times of technologies. The situation was exacerbated due to the number of small fleets (<20 trucks) in the industry. These small firms comprise over 90% of all U.S. trucking fleets (American Trucking Association, 2015). For smaller companies, high initial costs associated with technologies, coupled with the uncertainty of payback time represented a substantial market barrier. Another barrier to technology investment can be fuel costs, which fluctuate, creating more uncertainty.

From the supply side, truck manufacturers–although large corporations–sell products with relatively low sales volumes. This means that research and development costs are spread over a far smaller number of products than is the case with passenger vehicle manufacturers. Furthermore, trucks are a costly investment with complex specifications and numerous customization options. These variables raise the risks for OEMs to change proven model designs where new design flaws would make it even more costly for the manufacturers and for their customers. As a result, manufacturers are cautious in developing new technologies absent strong market demand signals. This creates a “chicken and egg” problem for truck manufacturers.

#### 3.2.2. Information gaps and process barriers

Though transportation is the largest end-use sector emitting carbon from fossil fuel combustion, and carbon is a common metric for measuring sustainability, within transportation the freight industry did not have a common method for measuring and reporting carbon emissions. Prior to the implementation of SmartWay in the U.S., shippers and carriers had not endeavored to collaborate on a uniform, reliable, transparent or verifiable system to exchange information and data about the environmental performance of goods movement, let alone even come to an agreement on how to do so. The trucking industry in particular is intensely competitive and sees very thin operating margins. Thus carriers were not only protective of disclosing performance information to their shipper clients, but peers and competitors in the freight sector also resisted sharing data that might adversely affect their competiveness.

As a result, carriers lacked the ability to benchmark their own environmental performance against their competitors with a common set of performance metrics. Drivers also lacked information about how to modify their driving behavior to improve fuel efficiency. Shippers lacked credible data they could use to understand carrier environmental performance and enable decision making to improve their freight emissions footprint.

#### 3.2.3. Communication barriers

Freight industry shippers and carriers have competing business objectives. Carriers want to maximize their profitability and competitiveness while shippers are focused on reducing transportation cost while ensuring timely, secure and reliable freight services. These competing objectives pose a challenge for fostering collaboration, sharing information and establishing trust.

Though several critically important industry and trade associations exist, these organizations serve the needs of each of their unique carrier, shipper or logistics management constituencies. In this business environment there is a real need for a platform where the two parties can find a common ground to collaborate and achieve success. There was no credible, neutral third party or “honest broker” which could facilitate a collection and transfer of data which could help industry to meet its sustainability goals. Industry leaders recognized the need and acknowledged opportunities to collaborate together to address their common challenges. Some began to turn to EPA to explore how this work could be done.

### 3.3. Addressing barriers and gaps through establishment of a voluntary policy model

#### 3.3.1. Addressing technology barriers

A lack of credible information about technologies inhibits the uptake in new fuel-saving products (Roeth et al., 2013, p. 5). Early on, EPA recognized the need to provide credible and neutral assessments of fuel-saving technologies. In the early 2000s, EPA commissioned a study to assess various options for improving ground freight fuel efficiency (ICF International, 2002). Following this study, EPA created a SmartWay technology assessment program. The program collaborated with manufacturers, testing facilities, end users and other stakeholders to update and refine test methods for assessing truck equipment (tires, aerodynamic fairings, idle reduction devices) and vehicles (class 8 trucks and box trailers). The program established a scope of technologies that could be verified (based on relative contribution to fuel savings and ability to retrofit). It established baseline values for representative performance, and improvement targets for fuel savings and reductions in CO_2_ emissions. It also established a protocol and eligibility criteria for “SmartWay” product verification.

Additionally, the program established a “SmartWay” designation for class 8 highway trucks and large box trailers. These vehicles could be designated as “SmartWay” if they met certain equipment and environmental performance eligibility criteria.

#### 3.3.2. Addressing information gaps and data barriers

Working closely with its industry partners and stakeholders, EPA developed calculator tools to track and measure the most common metrics used to assess freight sustainability: fuel consumption and carbon emission outputs (emissions for criteria air pollutants also are provided, though they are not commonly used for sustainability reporting). These metrics are summarized in partner-specific reports.

The calculator tools for carriers, shippers, and logistic firms were designed under the same platform and peer-reviewed by government, industry and academia. They provide uniformity, transparency, and can be accessed by the industry to communicate freight efficiency and sustainability within the intricately linked supply chain.

These assessment tools draw upon peer-reviewed methodologies and equations used by EPA. The criteria emissions or emissions factors as calculated by the SmartWay performance assessment tools are derived from EPA’s national Motor Vehicle Emission Simulator modeling system which estimates emissions for mobile sources (MOVES, 2015). The tools provide a comprehensive and granular level of assessment and tracking of carbon and criteria emissions.

The tools mirror relationships and information streams that are typical in the freight industry. The tools use data sources and descriptors already widely recognized and used by industry. For example, a carrier must submit fuel tax records to IFTA (International Fuel Tax Agreement); most use some form of GPS mileage software. Both these sources can be used to document the activity data (fuel use, miles traveled) required to complete a SmartWay assessment. And, to influence decision-making, EPA aligned the reporting categories with the same level at which the freight industry purchases and delivers freight transportation–the trucking fleet level. The program employs more than twelve truck fleet categories generally aligned with the heavy-duty trucking sector for grouping about three thousand SmartWay truck fleets, as shown in Table 1.

Each fleet category represents a unique but similarly grouped operation type. This detailed segmentation of truck fleets into a manageable number of categories enables appropriate, equitable comparison of fleets with their industry peers–an essential component for benchmarking environmental performance. This is because similar truck configurations and operational types can experience the benefits of technical and operational approaches in roughly comparable ways, making comparing efficiency and performance more equitable and actionable.

**Fig. 1.**
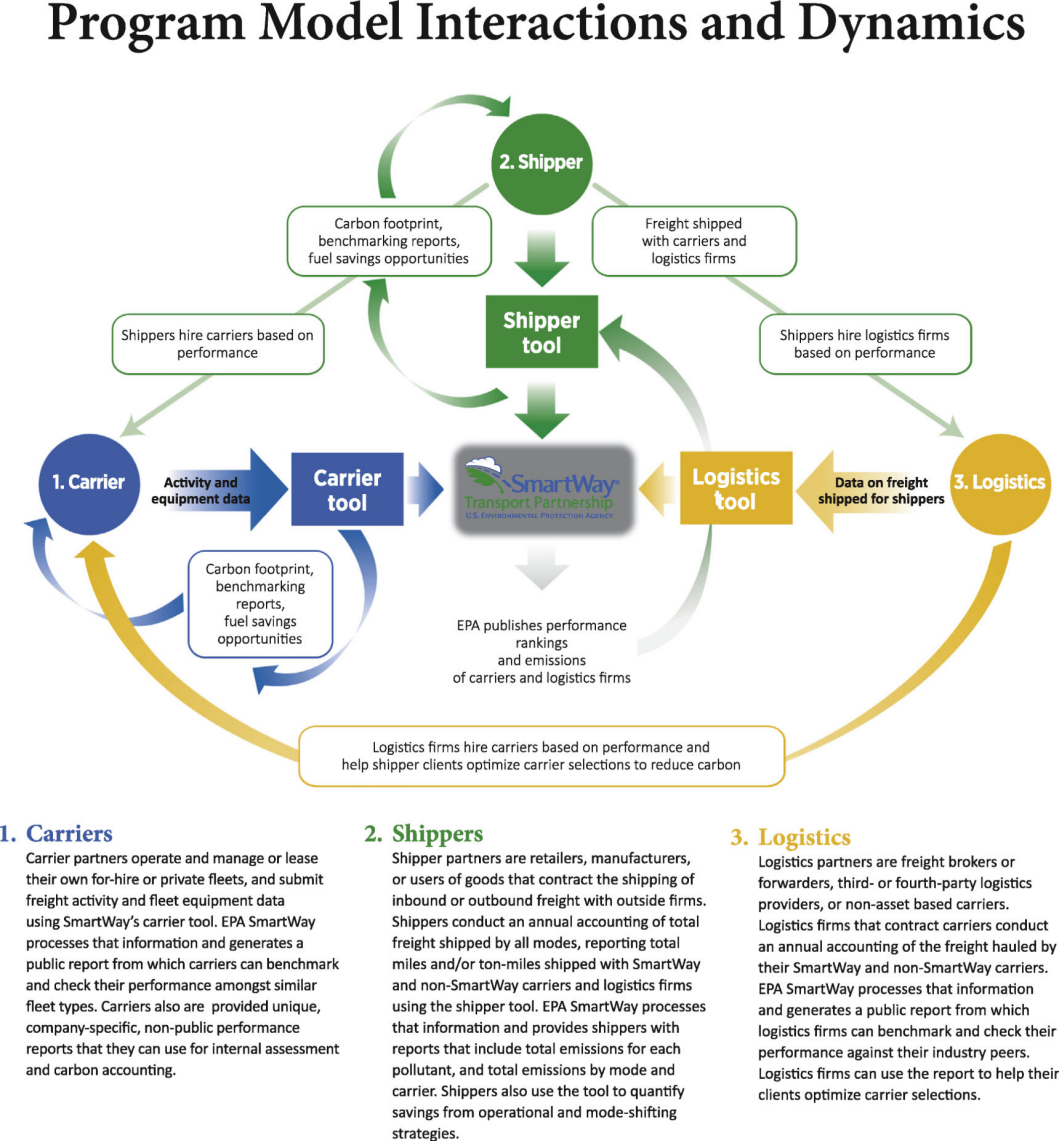
Data flows in SmartWay benchmarking and reporting process.

Finally, EPA organized the tools to mirror the hierarchy in the freight industry: carriers report first, then the data feeds into multimodal and logistics tools, which in turn are used to complete the shipper tool, reflecting the hierarchy of the freight industry. The data flows are presented in Fig. 1.

##### 3.3.2.1. Assessing performance

Emissions outputs from carrier tools are the basis for the SmartWay ranking system that the program developed in close collaboration with its partners and other stakeholders. Tool data is uploaded to an EPA-developed and maintained database, where it is stored and used to compute performance ranges for each partner category and subcategory. This in turn enables a partner to pull up prior year information to benchmark performance year over year; it also enables EPA to reduce some manual input, if the data is unchanged (e.g., contact information, number and model year of truck).

**Fig. 2.**
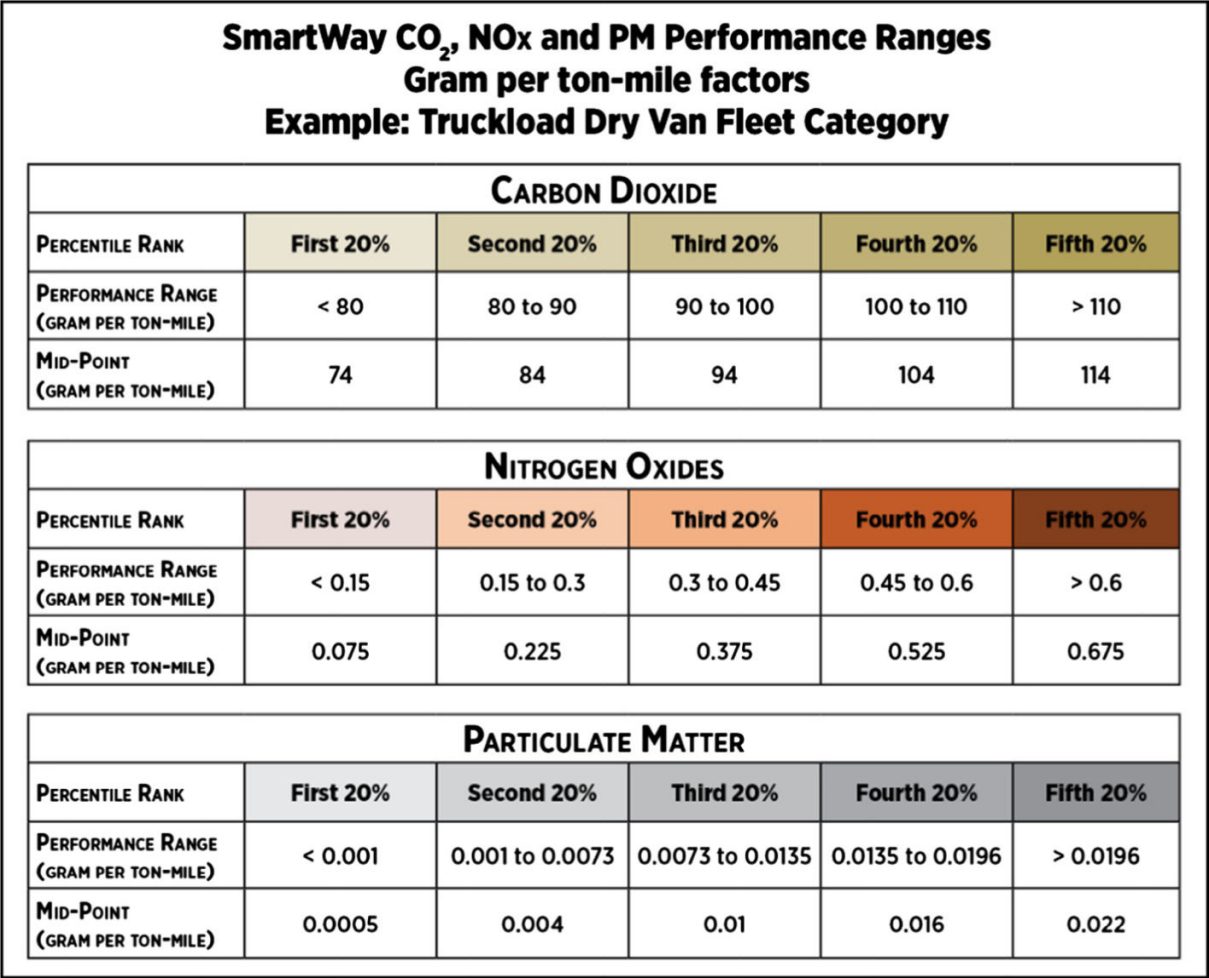
SmartWay environmental performance metrics expressed on gram per ton-mile basis and organized into five ranges, inclusive of mid-points for truckload dry van fleet types (2015 reporting year).

**Fig. 3.**
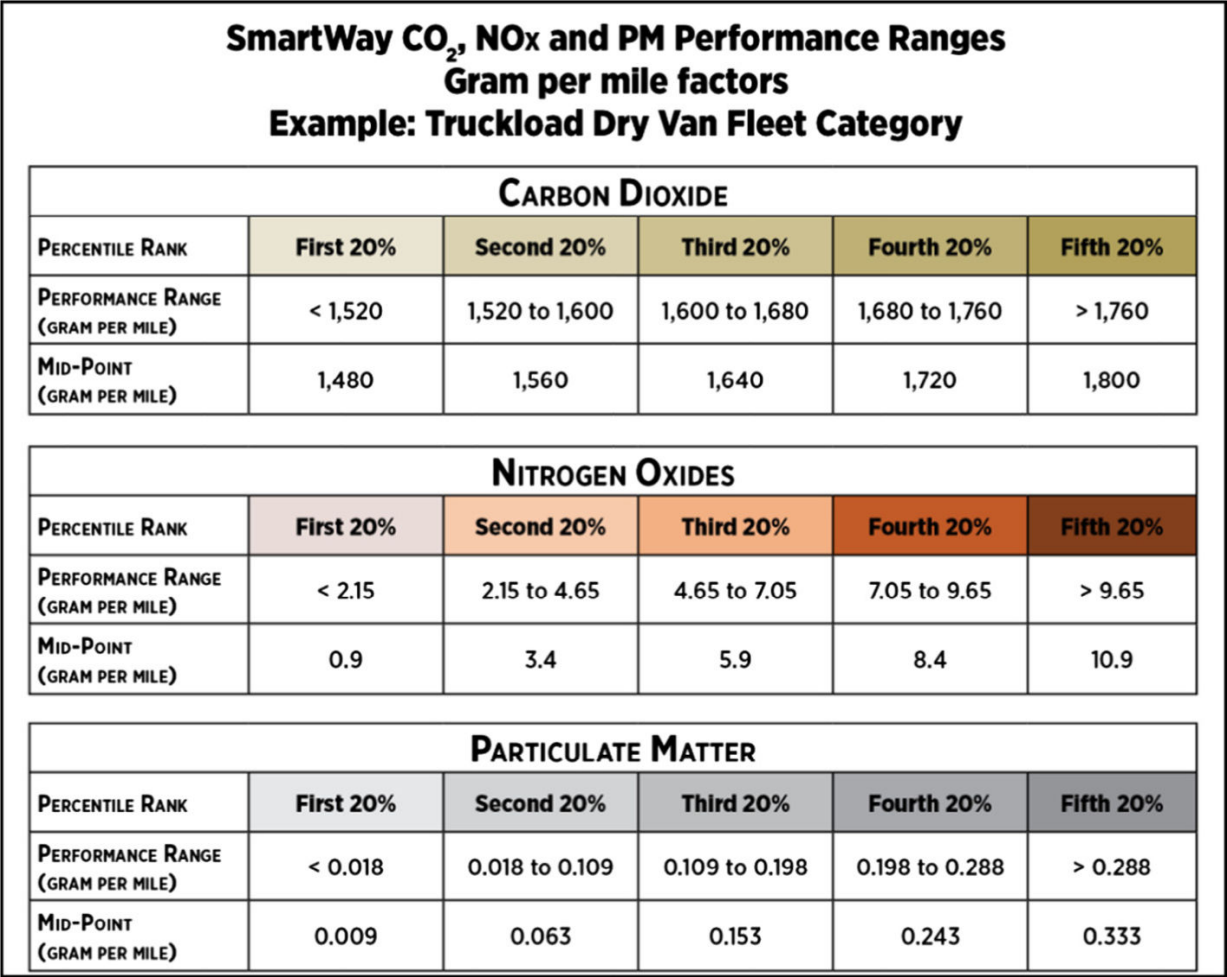
SmartWay environmental performance metrics expressed on gram per mile basis and organized into five ranges, inclusive of mid-points for truckload dry van fleet types (2015 reporting year).

As seen in Figs. 2 and 3, for each approved tool, the database calculates six emissions metrics, i.e., gram per ton-mile and gram per mile for carbon dioxide (CO_2_), nitrogen oxides (NOx) and particulate matter (PM), for each fleet category. Using the Truckload Dry Van and the gram per ton-mile or gram per mile metric as an example, the database aggregates the gram per ton-mile or gram per mile emissions for all truck fleets in the Truckload Dry Van category and generates performance ranges from 1 to 5, representing the lowest to the highest emitting fleets. This process is applied for each metric and fleet category.

##### 3.3.2.2. Maintaining data transparency and accessibility

SmartWay posts performance ranking reports on its website. To preserve confidentiality, SmartWay does not publish the absolute emissions numbers, but rather the mid-point performance range emissions, of each carrier. As shown in Table 2, the information on the performance ranking report can be used several ways to drive improvements or business decisions for partners of each type. Shippers are equipped with necessary data to make informed decision in its carrier selection, while the carriers can gauge performance relative to their industry peers, thus setting realistic targets for improving, such as investing in verified technologies or applying best practices in fleet operations. For logistics service providers, this information is used to offer fuel efficient transportation services to its customers, which are often the shipper companies.

##### 3.3.2.3. Maintaining data quality and integrity

Data quality is fundamental to credible and accurate benchmarking which can inform decision making and lead to emissions reductions. This strengthens brand integrity, adding further value for companies that participate in SmartWay. To maintain data quality, SmartWay has produced many resources and documents, and also embedded many help, guidance and data checks throughout tool screens to assist partner’s understanding of the required data parameters which SmartWay uses to calculate annual emissions. As the partners enter information, many input parameters are instantly validated. Pop-up window guidance assists partner data entry along this process. Once partners complete and submit tools to the program, SmartWay partner account managers review each tool. These account managers are available to partners to answer questions and provide assistance.

#### 3.3.3. Addressing communication barriers

SmartWay also was designed to help address the challenges posed by the industry’s business environment, where there exists competing goals, a need for a credible and qualified information source and neutral platform for communications. For example, in support of its technology verification program SmartWay developed communication processes, channels and materials to disseminate information on the emissions and fuel efficiency benefits of equipment components and the tractors and trailers qualified for SmartWay designation. This technology verification, designation and technology information was broadly and publicly distributed to a wide range of stakeholders with an interest in freight. This included frequent presentations at and participation in industry events focused on technologies and vehicles including industry trade shows and conferences, and academic and professional conferences and meetings.

The program is accessed as a credible and reliable information source by industry, other government agencies, academia, non-governmental organizations and civil society. SmartWay helps bridge the knowledge gap between freight professionals, technology providers and manufacturers across the board. SmartWay maintains an interactive website, tutorials; hosts a quarterly webinar program, workshops; and provides other opportunities for industry experts to share strategies and best practices that organizations can use to improve freight efficiencies and data quality. Through these communication and outreach programs SmartWay has helped facilitate industry wide efficiency gains and emissions reductions.

## 4. Discussion

### 4.1. Influence of the SmartWay model on shipper behavior

**Fig. 4.**
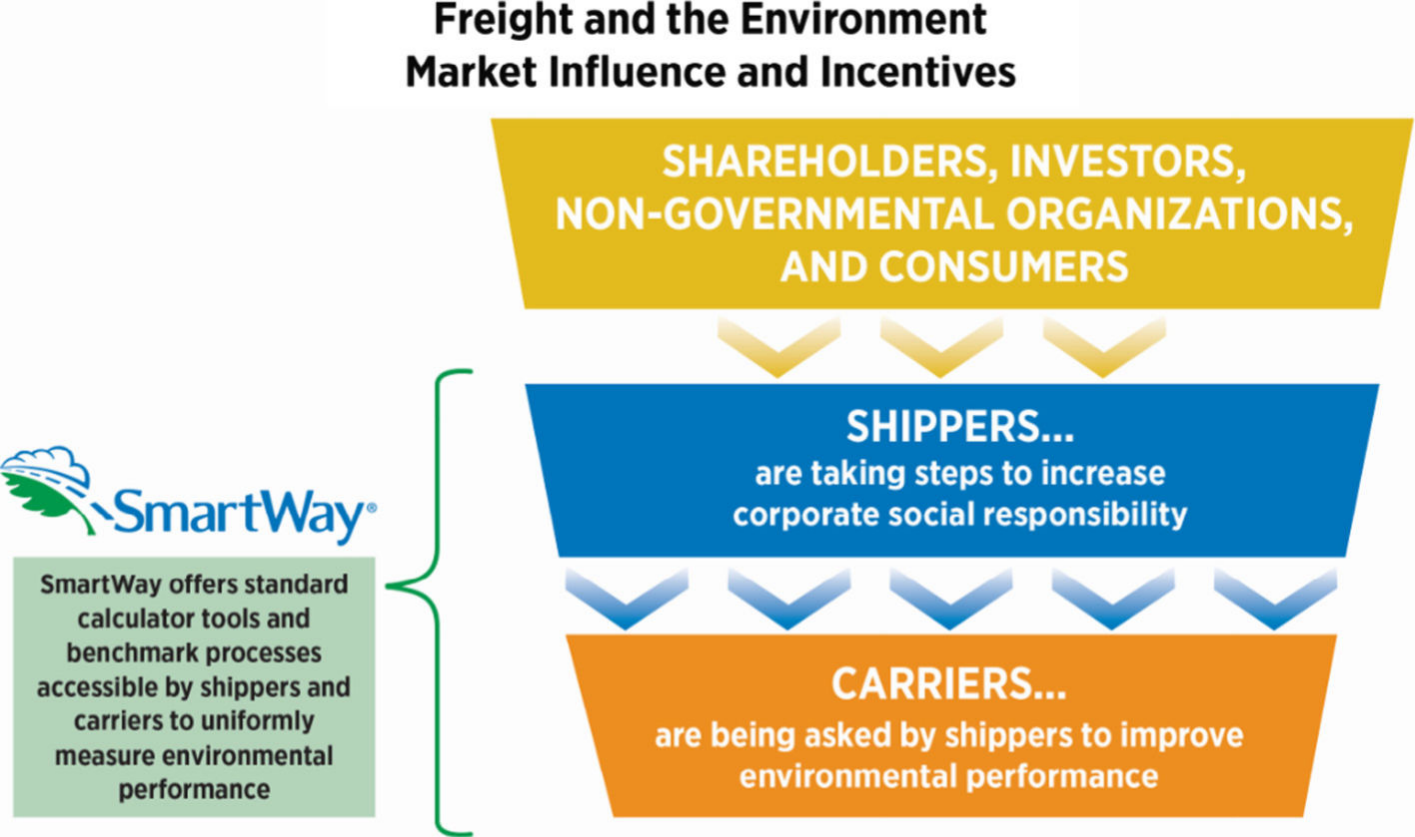
Schematics of the market dynamics.

Defined by Portney, “market-based” approaches to environmental policy utilizes the powerful advantages of markets in service to the environment (2007, p. 225). The SmartWay program design applies this concept to practice. As depicted in Fig. 4, the SmartWay voluntary policy model influences shipper and carrier interactions by leveraging the dynamics between shippers (as customers to their carriers) and carriers (as suppliers to their shippers).

As public-facing entities that service consumers, shippers have come under increasing pressure to improve the sustainability of their supply chains. This pressure comes from their customers, shareholders and investors, and non-governmental organizations. In response, shippers are measuring and reporting on their progress through corporate sustainability reports and disclosing their emissions and other sustainability metrics to various reporting frameworks, such as the Global Reporting Initiative and Carbon Disclosure Project, among others.

Shippers can improve the environmental performance of their freight operations by selecting carriers with lower emissions. This decision making is enabled by the SmartWay data and emissions ratings. Applying those data to other decision points, i.e., cost, reliability, service, etc., gives shippers another key factor in selecting top carriers. This mechanism leverages the market and drives carriers to accelerate efficiency efforts and be more transparent about their performance and emissions. When shippers increasingly require or prefer SmartWay registered carriers, more carriers are driven to register in the program and report performance. When more shippers require or prefer higher performing carriers (to reduce their supply chain emissions) this drives carriers to invest in more fuel saving strategies and technologies.

#### 4.1.1. Strategic shipper and carrier interaction

The SmartWay voluntary policy model influences shipper behavior at what is one of the most crucial decision points in the supply chain–carrier selection.

As mentioned previously, SmartWay creates a credible source of key environmental performance data for which shipper companies can use as a criterion for decision making and in their own sustainability benchmarking and reporting. Relating to the “market-based” definition, the SmartWay interventions center on facilitating supply chain benchmarking–an essential step towards improving environmental performance (O’Rourke et al., 2012).

Specifically, SmartWay provides standardized assessment tools for partners with a consistent set of metrics, terms, and methodologies, which help reduce variability and ensure continuity of the data from one year to the next. The methodologies, approaches and sources for the data are extensively documented in a number of technical reports to help partners and the supply chain community alike understand freight environmental performance. By presenting the data in a uniform, transparent, and readily accessible manner and into fleet categories that mirror how freight services are purchased (see Table 1), all carriers are assessed with the same yardstick, thus enabling equitable comparison of carriers. To ensure the integrity and the quality of the data, SmartWay collaborated with industry partners who demonstrated consistent program compliance with quality tools. Through partner site visits and interviews, the program identified and synthesized the industry best practices beginning from key data collection, aggregation, management to verification. These best practices are assembled in a document that was extensively reviewed by partners and industry experts (U.S. EPA, 2013). All partners can access this document to help guide and educate themselves on the data processes and practices for obtaining environmental performance. Table 3 lists a number of SmartWay interventions, objectives, and the market outcome.

While the extent of these interventions cannot be readily quantified, the program has observed significant and consistent behavioral changes from SmartWay shippers. Many shippers now make SmartWay registration a requirement for the carriers they hire (Kilcarr, 2015; Groeber, 2013). A small but growing subset of shippers are going beyond a simple requirement for SmartWay enrollment to basing business decision on carrier performance (U.S. EPA, 2015b). For example, in addition to making SmartWay registration a preference in their RFP (Request for Proposal) processes, some shippers now offer varying incentives for their carriers. These incentives range from offering fuel subsidies or more attractive shipping and receiving hours to carriers who participate in the SmartWay program or creating a performance-based rate structure that encourages carrier performance improvement (Tan and Blanco, 2009; U.S. EPA, 2015b). Also, many new carriers join the SmartWay program due to the recommendation of the shippers for which they transport freight (Ellram and Golicic, 2015; Wolmarans et al., 2014).

### 4.2. Influence of the SmartWay model on technology choices

#### 4.2.1. Manufacturer behavior – technology development

Technology verification is essential to provide credible ratings of technologies so that the marketplace has confidence they will perform as claimed. The SmartWay program influences technology advancement by manufacturers to achieve greater fuel savings and CO_2_ reduction potential.

The SmartWay technology assessment program provided manufacturers with a standard test protocol and methodology to objectively and credibly verify fuel savings of multiple categories of technologies: tires, aerodynamics and idle reduction technologies. That protocol became the basis for listings of verified technologies from which partners can learn about as options to improve performance (“SmartWay Opportunities for Manufacturers,” 2015; “SmartWay Technology,” 2015).

This list of technologies also became a basis for the standard equipment configurations known as the “designated SmartWay” tractor and trailer, and the “SmartWay Elite” trailer. These equipment configurations are available as retrofits for legacy vehicles and as standard packages for new trucks and trailers. By serving as a neutral third party that provides performance-based specifications and test protocols, SmartWay helped OEMs and their suppliers to innovate and demonstrate the fuel savings benefits of their equipment. Importantly, this facilitated fleet investments and helped alleviate the market barriers that existed.

Over the years, the program observed a change in behavior by manufacturers in the following aspects. For example, when EPA launched the SmartWay truck designation, every major truck manufacturer quickly offered one or more Class 8 over-the-road (OTR) models that qualified for this designation. Similarly, when EPA launched the SmartWay trailer designation, every box trailer manufacturer soon offered one or more trailer models that qualified for this designation. Concurrently, there has been a rapid growth in the number of verified tires, aerodynamic equipment, and anti-idling devices. Table 4 shows the growth in uptake of these technologies.

As another indication of market transformation, the industry now approaches SmartWay with requests to expand the list of verified technologies and designated truck types (e.g., day cab, tire inflation systems). As a result of this industry demand, the program now includes low rolling resistance retread tires in SmartWay tire verification which began in 2012. To encourage performance improvements, the program continued advancement in verified technologies which led to a new SmartWay “Elite” trailer mark that was launched in 2014, moving the bar from a 5 to a 9% reduction in fuel consumption from trailer aerodynamic devices.

#### 4.2.2. Carrier behavior – technology selection

The SmartWay program provides the market with reliable information on the efficiency and emissions benefits of truck equipment and technologies. Through EPA’s SmartWay verification program, lists of tractor and trailer equipment and technologies, along with a description of their expected fuel savings and emissions benefits are now publicly and widely available. By serving as a credible source for information on tractor and trailer equipment efficacy, the SmartWay voluntary policy model influences carrier behavior at an important juncture that positively effects the environmental performance of the supply chain: vehicle technology selection. SmartWay has given fleet owners and operators greater certainty and the data needed to identify, compare and make equipment investments decisions that have helped improve fleet performance, by lowering emissions and fuel use.

Similarly, fleets can now easily and quickly identify the cleanest and most fuel efficient tractor and trailer models available in the market through the SmartWay designation program. When the SmartWay tractor program first launched in 2007, every Class 8 OTR truck manufacturer offered at least one designated tractor model. Today, the six Class 8 OTR truck manufacturers have expanded the selection of their top performing tractor models that meet the performance criteria necessary to qualify for the SmartWay designation, providing fleets with more options to invest in more sustainable fleets.

EPA SmartWay also influences and informs the market on equipment and technology upgrades in other ways that has contributed to a more advanced national trucking fleet. The partnership has published peer-reviewed papers and technical fact sheets that describe the testing protocols, performance criteria and test data that it uses to ensure that vehicles, equipment and technologies will help fleets improve their efficiency and reduce emissions (Bachman et al., 2005; Bachman et al., 2006; Bachman et al., 2014; Waltzer et al., 2015). Through its verification and designation programs, and by actively contributing to trade, industry and academic journals EPA SmartWay has advanced the freight industry’s understanding of the benefits of technology adoption and the economic value of sustainability.

SmartWay invites and encourages fleets to provide feedback on their experience with new technology and operational strategy investments. Several case studies and profiles detailing the benefits and outcome have been published, furthering continued support and improvement. Several large SmartWay partners also conduct their own independent technology development and testing programs, and share best practices to further spur industry progress. This has led to broader widespread adoption of these technologies during the past decade.

Many other companies that have joined SmartWay adopt SmartWay verified technologies for certain applications and invest in SmartWay designated tractors and trailers, as their long-haul combination truck fleets turnover. As SmartWay grows, the program attracts new entrants. This has a potential effect of lowering overall program performance until these carriers improve. Despite this trend, the program is contributing to environmental improvements. As shown in Table 5, SmartWay carriers in the truckload dry van and refrigerated fleet categories collectively improved performance by 3.4 and 2.5%, respectively, in the past two years as measured by gram/ton-mile, a metric widely used by the freight industry to gauge efficiency.

### 4.2.3. Regulatory complement

In addition to influencing technology advancement and adoption by OEMs and carriers, respectively, the technology approaches developed through SmartWay to verify truck technologies have been used to support U.S. national and state policies and regulations. Most notably, in 2011, the U.S. Environmental Protection Agency and the Department of Transportation’s National Highway Traffic Safety Administration issued a first-ever regulatory program to reduce GHG emissions and improve fuel efficiency of heavy-duty trucks and buses. This national heavy-duty program drew heavily from the SmartWay verification program to identify specific tractor design features, equipment technologies, and test methods used to reduce emissions and improve freight performance. Similarly, the testing protocols and results from SmartWay’s verification program are widely cited by the National Research Council (NRC) in reports used by EPA and DOT in developing their joint GHG emissions and fuel consumption standards (NRC, 2012; NRC, 2015).

Technical results reported by the SmartWay program also have garnered attention from regions and states. California’s Global Warming Solution Act of 2006 resulted in subsequent and numerous regulations that utilize key elements of the SmartWay verification and vehicle designation program, requiring most truck carriers operating in the state to upgrade their fleets with aerodynamic technologies, low rolling resistant tires, and other equipment verified by SmartWay (2006).

To reduce diesel emissions from aging diesel fleets, many state grants focused on retrofitting equipment with SmartWay technologies, such as idle reduction devices (Diesel Emissions Reduction Act of 2010, 2011). Through its verification program, SmartWay has visibly complemented the standard-setting aspect and goals of regulation. In other ways, the design of the program has positively influenced the relationship between the trucking industry and regulatory agencies, such as the EPA. And by working together SmartWay and its partners have also helped build trust between government and industry, through developing a shared understanding of the importance of sustainability to the health of the industry.

As more fleet owners and operators gain experience and learn how technology can help improve their performance and become more competitive, they are more willing to collaborate and are better positioned to adjust to emerging policies and regulations that are imposing new standards on the industry.

### 4.3. A global green freight template

Freight transport is a cornerstone in today’s globalized economy. Freight transport and logistics systems enable economic growth in all parts of the world, and have especially impacted developing nations and regions with export driven economies. As the economies in these nations improve, so too does the demand for better and more efficient means to move and transport goods.

Policy makers in both developed and developing countries are now looking for options to improve efficiency and mitigate emissions from freight activity, and the SmartWay voluntary partnership model has played an increasingly important role. SmartWay’s influence is especially apparent in North America, where both Canada and Mexico have adopted key elements of the program.

In 2012 a letter of agreement was signed by U.S. and Canadian officials, whereby Canada fully integrated the SmartWay brand, benchmarking tools and performance metrics into its freight transportation program (SmartWay Canada, 2015). Today the two nations fully cooperate and work with one another through SmartWay to address the carbon emissions that are generated by goods movement both within their borders and across the border.

Environmental officials in Mexico adopted the SmartWay model’s basic carbon assessment and measurement tools in 2010, and are exploring further integration. The U.S. EPA, Natural Resources Canada, and Mexico’s Transport Limpio are working to extend their collaboration on freight-related matters by using the SmartWay platform in their voluntary outreach to shippers and carriers that move goods across North America.

In 2008, the Environmental Protection Bureau for Guangzhou, China reached out to EPA SmartWay for technical assistance in designing a “green” truck project in the industrialized Guangdong province (World Bank, 2010). The Bureau employed SmartWay verified technologies (idle reduction equipment, tires and aerodynamics) in a comprehensive demonstration program. The results of this demonstration project were used by the Chinese Ministry of Transport in launching its nationwide “China Green Freight Initiative,” a public-private program aimed at improved fuel efficiency and lower CO_2_ emission from road freight transport, which has replicated many key elements from the SmartWay partnership model (2015).

At the international level, the United Nations Environment Programme’s Climate and Clean Air Coalition has identified SmartWay as a good model for the growing ‘green freight movement’ (Punte and Muncrief, 2015). EPA SmartWay has participated and shared its experience at several coalition workshops in Brazil, Vietnam and Chile. The International Council for Clean Transportation funded a project to create a “How to Develop a Green Freight Program” training manual, based almost exclusively on the SmartWay voluntary partnership model (U.S. EPA, 2014). The manual has been translated into five languages including, English, Spanish, Chinese Mandarin, French and Portugese.

Freight transport is an increasingly important economic sector, fueled by economic growth, globalization of markets and urbanization (Smart Freight Centre, 2015). International trade is expected to continue to grow in the global economy. International standards facilitate trade, and similarly a global system for monitoring and calculating freight emissions will facilitate a stronger, more efficient global green freight network.

In an effort to create a universally adopted framework for calculating freight emissions, the Global Logistics Emissions Council (GLEC) has developed a methodology (2015). To meet its goal for a harmonized freight emissions calculation, GLEC has built upon existing models, including SmartWay. In its work with GLEC and other nations SmartWay has positively contributed to establishing a harmonized framework that will allow for an accurate comparison of emissions, smarter decision-making and effective emission reduction strategies across national and regional borders.

Overall, these international agreements and initiatives illustrate SmartWay’s significant influence on global policy makers and governments.

## 5. Conclusion

As the world’s nations increasingly recognize the risk of climate change and seek solutions, there is a growing understanding of freight’s contribution to climate change and public health. Identifying sustainable approaches to address freight’s emissions impacts is a critical, yet complex, challenge that confronts both the private and public sectors, neither of which can accomplish this objective on its own (Wolmarans et al., 2014).

Though the U.S. recently established a mandatory Heavy-Duty National Program that sets greenhouse gas and fuel efficiency standards for new medium- and heavy-duty vehicles, these new standards will take several years before fully phasing in, and even longer until the legacy fleet has completely turned over. For this reason, there is a need for voluntary partnerships, like SmartWay, that more immediately address existing emission sources and influence change in the freight industry. Importantly, these changes are linked to the behaviors of the many players, activities, decisions and relationships of those involved in moving freight.

The voluntary SmartWay partnership model has brought these players closer together by creating a platform where shippers and carriers can openly communicate over emerging issues around climate change, and related environmental impacts. By providing the industry with neutral testing protocols and independent performance data, SmartWay has helped to spur innovation, greater adoption, and investment in technologies that save fuel and reduce greenhouse gas emissions. Voluntary programs like SmartWay can also serve as a test bed where public policy and free market trends can be integrated to help prevent adverse unintended consequences that are sometimes posed by regulation and strict mandates.

The program’s carbon assessment and benchmarking tools give carriers a transparent and uniform system to measure and benchmark freight emissions, differentiate themselves, and respond to shipper requests. Supply chain performance is coming under increasing scrutiny and shippers are starting to use SmartWay data to report freight emissions in their corporate reports and to independent agencies, such as the Global Reporting Initiative and Carbon Disclosure Project.

SmartWay’s influence is also apparent in the overall performance of its partnership base. Collectively, for the past ten years, as new companies join the partnership, the program continues to demonstrate progress. The program grew from 15 Charter Partners to over 3000 partners today. Together, these partners have prevented cumulatively 72.8 million metric tons of CO_2_, 1.46 million tons of nitrogen oxides, and 59 thousand tons of particulate matter (U.S. EPA, 2016).

This progress reflects a continual improvement among those carriers and shippers that voluntarily join SmartWay. Many partners that have participated in the partnership for several years were early adopters of SmartWay verified equipment and designated vehicles, and are now steadily operating in the top range of performance. These partners are important to the program and the freight industry. Through SmartWay, they have an opportunity to demonstrate leadership, share best practices and encourage other partners to improve.

Internationally, the SmartWay model has been widely accepted by the freight industry because it provides a comprehensive approach to improving freight’s environmental performance, and consistent metrics that help level the playing field. Elements of the model are currently being integrated into a global accounting system under development by the Global Logistics Emissions Council. Individually, nations also have adopted key elements of the partnership model as they begin to tackle freight’s environmental challenges.

Public sector policy makers are increasingly looking at the opportunities presented by implementing programs like SmartWay. Challenges associated with protecting local communities from growing sources of transportation emissions or national global climate concerns can be addressed by using these programs to drive emission reductions. Energy security can be enhanced by driving reductions in fuel use in this energy intensive and key economic sector. Economic development can be stimulated by fostering more efficient movement of goods, for trade and domestic consumption. Technology transfer can be accelerated by leveraging market mechanisms and driving the uptake of new technologies. Policy makers can serve the public and make a global impact by adopting programs like SmartWay.

The voluntary SmartWay partnership model has helped shippers and carriers recognize that freight emissions are a source of greenhouse gas emissions that can be reduced by removing market barriers, identifying economic incentives, openly communicating and working together. The model has helped industry and the U.S. EPA to effectively address emerging issues around freight transport and climate change. In the absence of regulation, and until such time as full compliance, the voluntary SmartWay model serves as an effective mechanism for achieving behavior change that can lead to emissions reduction and improved environmental performance in the freight sector.

## Figures and Tables

**Table 1:** SmartWay truck fleet categories according to industry usage.

Categories	General Characteristics
Truckload Dry Van	Movement of an enclosed, non-climate controlled rectangular trailer-load to a single customer
Refrigerated	Movement of cargo in an insulated box van under temperature control
Less than Truckload	Movement and consolidation of freight typically weighing less than 10,000 lb collected from various shippers
Drayage	Predominantly associated with port or rail head connections where freight is picked up and moved to another transfer facility or transport mode terminal
Flatbed	Movement of a trailer with an open body in the form of a platform without sides or doors
Expedited	Shipment of time-sensitive freight
Tanker	Movement of a trailer with a tank body for transporting bulk quantities of liquid or gas
Package Delivery	Residential or business package delivery and pickup consisting primarily of single or small groups of packages
Heavy Bulk	Movement of huge, heavy equipment or object, including hauls at or above the legal maximum weight limit (i.e., requiring permits)
Moving	Movement for residential or office moving activities
Specialized	Movement using specialized equipment such as hopper for grain or fertilizer, and for hauling livestock
Auto	Movement that uses a trailer designed to transport light-duty vehicles
Mixed	Less than 75% in any other category

**Table 2:** Purposes of SmartWay performance ranking report.

Partner type	How report is being used
Carriers	To gauge relative ranking to their SmartWay peers, thus setting targets for improving environmental performance
Shippers	To optimize or make informed decision in its carrier selection
Logistics service providers	To assist shippers in making informed carrier and mode selection

**Table 3:** SmartWay market interventions to partners and industry.

Market intervention	SmartWay objective and outcome
A public-private collaboration focused on developing and providing freight industry with consistent metrics, terms and methodologies for assessing environmental performance in the transportation of goods	**SmartWay Objective**: Establish carbon and criteria pollutants as key metrics for assessing efficiency and environmental performance in freight transport. Raise awareness around freight sustainability and encourage environmental and efficiency improvements
	**Outcome**: Freight carriers use carbon produced at the fleet level to inform market and benchmark their environmental performance
Calculation and ranking of carrier environmental performance as a neutral third party	**SmartWay Objective**: Provide freight marketplace with greater transparency and disclosure to help foster improved performance
	**Outcome**: Shipper’s carrier selection may now include environmental performance criteria in contract decisions (e.g., shipper requirement of SmartWay registration, application of various incentives in awarding contracts, etc.)
A testing protocol and program for heavy-duty trucks and aftermarket equipment to verify truck and equipment efficiency and environmental performance	**SmartWay Objective**: Serve as neutral third party and provide marketplace with greater certainty to invest in fuel-saving equipment with emissions benefits
	**Outcome**: Smartway tractor trailer designation and verification program are established, widely recognized and accessed by industry in acquisition and investment decisions

**Table 4:** Growth in number of SmartWay verified technologies and designated models.

Designated or verified technology types	2007	2015
Aerodynamics		
Fairings and skirts	7	70
Tail and gap reducer	4	13
Tires		
Steer	3	407
New drive	4	370
New trailer	4	241
Retread drive	n/a	38
Retread trailer	n/a	28
Idle reduction		
Electrified parking	2	7
Auxiliary power units and generator sets	6	25
Fuel operated heaters/Direct fire heaters (FOH/DFH)	1	11
Battery air conditioning systems	n/a	26
Thermal storage systems	n/a	2
Designated tractors	8	16
Designated trailers	n/a	*

* Expanded to include refrigerated trailers and added new “*Elite*” designation in 2014 for both dry van and refrigerated trailers.

**Table 5:** Percent emissions reduction by SmartWay fleets compared to 2012 levels.

% changes (gram/ton-mile)	2013	2014
Truckload dry van fleets	−1.6%	−3.4%
Refrigerated fleets	−1.6%	−2.5%
